# Childhood cancer and parental use of tobacco: findings from the inter-regional epidemiological study of childhood cancer (IRESCC)

**DOI:** 10.1054/bjoc.2000.1556

**Published:** 2001-01-01

**Authors:** T Sorahan, P A McKinney, J R Mann, R J Lancashire, C A Stiller, J M Birch, H E Dodd, R A Cartwright

**Affiliations:** Institute of Occupational Health, University of Birmingham, Edgbaston, Birmingham, B15 2TT; Paediatric Epidemiology Group, University of Leeds, 30 Hyde Terrace, Leeds, LS2 9LN; The Birmingham Children's Hospital NHS Trust, Steelhouse Lane, Birmingham, B4 6NH; Department of Public Health and Epidemiology, University of Birmingham, Edgbaston, Birmingham, B15 2TT; Childhood Cancer Research Group, 57 Woodstock Road, Oxford, OX2 6HJ; CRC Paediatric and Familial Cancer Research Group, Royal Manchester Children's Hospital, Stancliffe, Hospital Road, Manchester, M27 4HA; Leukaemia Research Fund Centre for Clinical Epidemiology at Leeds University, Margaret Smith Building, 30 Hyde Terrace, Leeds, LS2 9LN, UK

**Keywords:** childhood cancer, smoking, case-control study

## Abstract

Parental smoking data have been re-abstracted from the interview records of the Inter-Regional Epidemiological Study of Childhood Cancer (IRESCC) to test further the hypothesis that paternal cigarette smoking is a risk factor for the generality of childhood cancer. Reported cigarette smoking habits for the parents of 555 children diagnosed with cancer in the period 1980–1983 were compared, in two separate matched pairs analyses, with similar information for the parents of 555 children selected from GP lists (GP controls) and for the parents of 555 hospitalized children (hospital controls). When cases were compared with GP controls there was a statistically significant positive trend (*P* = 0.02) between the risk of childhood cancer and paternal daily consumption of cigarettes before the pregnancy; there was no significant trend for maternal smoking habit. When cases were compared with hospital controls there was a statistically significant negative trend (*P*< 0.001) between the risk of childhood cancer and maternal daily consumption of cigarettes before the pregnancy; there was no significant trend for paternal smoking habit. Neither of the significant trends could be explained by adjustment for socioeconomic grouping, ethnic origin or parental age at the birth of the child, or by simultaneous analysis of parental smoking habits. Relations between maternal consumption of cigarettes and birth weights suggested that (maternal) smoking data were equally reliable for case and control subjects, although comparisons with national data suggested that the hospital control parents were unusually heavy smokers. These findings give some support for the hypothesis that paternal cigarette smoking is a potential risk factor for the generality of childhood cancers. © 2001 Cancer Research Campaign http://www. bjcancer.com


British Journal of Cancer (2001) **84**(1), 141–146
© 2001 Cancer Research Campaign
doi: 10.1054/ bjoc.2000.1556, available online at http://www.idealibrary.com on IDEAL®

http://www.bjcancer.com

### Childhood cancer and parental use of tobacco: findings from the inter-regional epidemiological study of childhood cancer (IRESCC)

**T Sorahan[1], PA McKinney[2], JR Mann[3], RJ Lancashire[4], CA Stiller[5], JM Birch[6], HE Dodd[3] and RA Cartwright[7]**

[1]Institute of Occupational Health, University of Birmingham, Edgbaston, Birmingham B15 2TT; [2]Paediatric Epidemiology Group, University of Leeds, 30 Hyde Terrace, Leeds LS2 9LN; [3]The Birmingham Children's Hospital NHS Trust, Steelhouse Lane, Birmingham B4 6NH; [4]Department of Public Health and Epidemiology, University of Birmingham, Edgbaston, Birmingham B15 2TT; [5]Childhood Cancer Research Group, 57 Woodstock Road, Oxford OX2 6HJ; [6]CRC Paediatric and Familial Cancer Research Group, Royal Manchester Children's Hospital, Stancliffe, Hospital Road, Manchester M27 4HA; [7]Leukaemia Research Fund Centre for Clinical Epidemiology at Leeds University, Margaret Smith Building, 30 Hyde Terrace, Leeds LS2 9LN, UK

**Summary** Parental smoking data have been re-abstracted from the interview records of the Inter-Regional Epidemiological Study of Childhood Cancer (IRESCC) to test further the hypothesis that paternal cigarette smoking is a risk factor for the generality of childhood cancer. Reported cigarette smoking habits for the parents of 555 children diagnosed with cancer in the period 1980–1983 were compared, in two separate matched pairs analyses, with similar information for the parents of 555 children selected from GP lists (GP controls) and for the parents of 555 hospitalized children (hospital controls). When cases were compared with GP controls there was a statistically significant positive trend ($P = 0.02$) between the risk of childhood cancer and paternal daily consumption of cigarettes before the pregnancy; there was no significant trend for maternal smoking habit. When cases were compared with hospital controls there was a statistically significant negative trend ($P < 0.001$) between the risk of childhood cancer and maternal daily consumption of cigarettes before the pregnancy; there was no significant trend for paternal smoking habit. Neither of the significant trends could be explained by adjustment for socioeconomic grouping, ethnic origin or parental age at the birth of the child, or by simultaneous analysis of parental smoking habits. Relations between maternal consumption of cigarettes and birth weights suggested that (maternal) smoking data were equally reliable for case and control subjects, although comparisons with national data suggested that the hospital control parents were unusually heavy smokers. These findings give some support for the hypothesis that paternal cigarette smoking is a potential risk factor for the generality of childhood cancers. © 2001 Cancer Research Campaign http://www. bjcancer.com

**Keywords**: childhood cancer; smoking; case-control study

Three large studies of UK childhood cancer risks in relation to reported parental use of tobacco (combined series of 5777 cases) are available from the Oxford Survey of Childhood Cancers (OSCC) (Sorahan et al, 1995; 1997*a*; 1997*b*). All three studies found no significant association with maternal smoking habit and highly significant positive trends with paternal smoking habit. Site-specific pooled estimates of risk (smokers vs non-smokers), obtained from all studies which provided information on childhood cancer risks in relation to paternal smoking, indicate that results for fathers cannot be easily dismissed as chance findings (all sites: four studies, relative risk (RR) = 1.26, 95% confidence interval (CI) = 1.13–1.40; leukaemia: seven studies, RR = 1.09, 95% CI = 1.03–1.15; lymphoma: five studies, RR = 1.21, 95% CI = 1.07–1.37; brain tumours: eight studies; RR = 1.18, 95% CI = 1.03–1.36; central nervous system tumours: five studies, RR = 1.11, 95% CI = 1.03–1.20) (Thornton and Lee, 1998).

Information on parental use of tobacco is also available for one further set of UK data, the Inter-Regional Epidemiological Study of Childhood Cancer (IRESCC) (Birch et al, 1985). These data have therefore been revisited to seek further information on the following hypothesis: paternal cigarette smoking is a risk factor for the overall grouping of all childhood cancers; maternal cigarette smoking is unimportant in this regard. There is a small degree of overlap between IRESCC cases and cases analysed in one of the OSCC reports (Sorahan et al, 1995).

*Received 9 August 2000*
*Revised 27 September 2000*
*Accepted 28 September 2000*

*Correspondence to:* T Sorahan. E-mail: T.M.Sorahan@bham.ac.uk

### MATERIALS AND METHODS

The IRESCC was established to investigate the role of possible aetiological factors in childhood cancer with particular emphasis on environmental exposures to the foetus and family history of diseases (Cartwright et al, 1984; Hopton et al, 1985; Johnston et al, 1986; McKinney and Stiller, 1986; McKinney et al, 1985; 1987; Hartley et al, 1988*a*; 1988*b*; Birch et al, 1990; Mann et al, 1993). Study design, control selection and data collection procedures have been published in some considerable detail (Birch et al, 1985); a summary is provided here. The survey sought to interview the parents of all 761 children resident in the Yorkshire, West Midlands and North Western Regional Health Authority areas who were first diagnosed with malignant disease (or allied condition) before their fifteenth birthday; diagnoses relate to the period January 1980 to January 1983. Children who were not living with their natural mother were excluded and a random sample of certain types of cancer were excluded to reduce the workload. Of the 615 cases eligible for interview, parents of 19 cases were not approached on the advice of their General Practitioner (GP) or consultant and parents of 41 cases declined to take part; interview data were obtained for 555 cases. It proved possible to approach most case parents soon after their children had been diagnosed with cancer.

For each case child with interview data, interview data were sought for two control children matched for sex and date of birth. One set of potential controls was selected from the practice lists of the case GPs, a second set of potential controls was selected from lists of acute surgical and accident cases from six large hospitals; hospital controls were drawn from hospitals in the same region as their respective cases. Control parents from each list were contacted in turn until one control family agreed to be interviewed. Interview data were obtained for 555 GP controls (400 first choices (72%), 111 second choices (20%) and 44 later choices (8%)). Interview data were obtained for 555 hospital controls (355 first choices (64%), 122 second choices (22%) and 78 later choices (14%)). Participation rates for approached parents were about 97% for cases, 74% for GP controls and 64% for hospital controls. Both parents were present at the interview for 59% of the cases, 50% of the GP controls and 50% of the hospital controls. Interviews were carried out by a small number of trained interviewers and all parents in any given case-control set were always interviewed by the same person.

For the purpose of this report, the micro-filmed interview records of all study subjects were reviewed and information on parental cigarette smoking habits was re-abstracted; the IRESCC computer files developed in the 1980s were in a machine-specific format not compatible with computers currently in use. The interview sought information for mothers on the question ‘Did you smoke before and/or during your pregnancy?’, and information for fathers on the question ‘Do you smoke or have you ever smoked?’. Positive responses for both parents were to be given in terms of ‘type of product’, ‘quantity and frequency’ and ‘dates’. Some interviewers collected detailed smoking histories with ‘dates’ given in terms of ages (e.g. 10 cigarettes per day (cpd) at ages 17–19, 10–20 cpd at ages 19–24, gave up when pregnancy was confirmed). Other interviewers collected summary information (e.g. before pregnancy 20–25 cpd, during pregnancy 10 cpd). All available information on consumption of cigarettes was re-abstracted and computerized in text form. The coding system applied to this analysis was that used in the earlier OSCC reports so that when the daily consumption of cigarettes was reported with upper and lower values, the upper value was selected. Parental age at the time of conception was calculated and the relevant daily smoking habits at this age were evaluated (smoking before the pregnancy). In addition the maternal smoking habit at the fifth month was also evaluated (smoking during the pregnancy). For those mothers providing summary smoking information (see earlier comments), the smoking habit ‘before the pregnancy’ was assumed to apply to the smoking habit at the time of conception and the smoking habit ‘during the pregnancy’ was assumed to apply to the fifth month of the pregnancy. Maternal smoking data had not been sought for six cases, and these cases together with their 12 controls have been excluded from the maternal analyses. The microfilm for one hospital control was not found. The smoking questions were left unanswered for one hospital control mother, 29 case fathers, 17 GP control fathers and 30 hospital control fathers; most of these fathers were not living with their children. Birth weight data were also re-abstracted for each child; information from obstetric notes or GP records took precedence over data obtained from mothers.

Case and control data relating to cigarette consumption were compared (with and without adjustment for other variables) by means of (multiple) conditional logistic regression using the EGRET program. Smoking habits of mothers and fathers were first analysed separately, then with additional adjustment for other variables (maternal age at the birth of the child: < 20 years, 20–24 years, 25–29 years, 30–34 years, 35–39 years, ≥ 40 years; paternal age at the birth of the child: same categories; socioeconomic grouping based on paternal occupation: professional and managerial, other white collar, industrial and manual, unemployed, not known; ethnic origin: Caucasian, Asian (Indian/Pakistani/Bangladeshi), West Indian, other). Finally, the smoking habits of both parents were analysed simultaneously. The purpose of the multiple regression analyses was to identify any independent effects of each smoking habit. The odds ratio was used to obtain estimates of relative risk (RR). Risks are shown relative to a baseline risk of unity for the non-smokers.

### RESULTS

Relative risks for all types of childhood cancers combined are shown by paternal cigarette smoking habits before the index pregnancy in Table 1. Cases are first compared with GP controls then with hospital controls. A significant positive trend ($P = 0.02$) is shown for smoking habit and childhood cancer risk when cases are compared with GP controls, with significantly elevated point estimates of relative risk for two of the intermediate smoking categories (10–19 cpd, RR = 1.63; 20–29 cpd, RR = 1.46). The highest relative risk (1.77) is shown for the highest smoking category (≥ 40 cpd) though this finding is based on relatively small numbers of case and control fathers. A non-significant negative trend ($P = 0.16$) is shown for the corresponding analysis with hospital controls; a significantly depressed point estimate of relative risk is shown for one of the intermediate smoking categories (30–39 cpd, RR = 0.45). Additional adjustment for paternal age at the birth of the survey child, socioeconomic category and ethnic origin had little material effects on these two sets of relative risks (not shown in Table); a significant positive trend with paternal smoking habit remained when cases were compared with GP controls ($P = 0.03$).

Relative risks for all types of childhood cancers combined are also shown by maternal cigarette smoking habits before the pregnancy in Table 1. A non-significant positive trend ($P = 0.53$) is shown for smoking habit and childhood cancer risk when cases are compared with GP controls, albeit there are significantly elevated point estimates of relative risk for the two lowest smoking categories (< 10 cpd, RR = 1.77; 10–19 cpd, RR = 1.51). A highly significant negative trend ($P < 0.001$) is shown for the corresponding analysis with hospital controls, with significantly depressed relative risks for the two highest smoking categories (20–29 cpd, RR = 0.64; ≥ 30 cpd, RR = 0.18). Additional adjustment for maternal age at the birth of the survey child, socioeconomic category and ethnic origin had little material effects on these two sets of relative risks (not shown in Table); a significant negative trend with maternal smoking habit remained when cases were compared with hospital controls ($P = 0.003$). Simultaneous analysis of parental smoking habits left a significant positive trend ($P = 0.03$) between childhood cancer risk and paternal smoking habit when cases were compared with GP controls and a significant negative trend ($P < 0.001$) between childhood cancer risk and

 © 2001 Cancer Research Campaign

**Table 1** Childhood cancer risks by parental cigarette smoking habits before the pregnancy (time of conception): IRESCC data, 1980–1983 diagnoses

| Parental smoking habit | Cases | GP controls | Hospital controls | Childhood cancer risk: Cases vs GP controls | | Cases vs Hospital controls | | Mean birthweight (ounces) | | |
|---|---|---|---|---|---|---|---|---|---|---|
| | (*n*) | (*n*) | (*n*) | RR | 95% CI | RR | 95% CI | Cases | GP controls | Hospital controls |
| **Fathers** | | | | | | | | | | |
| Lifelong non-smoker | 184 | 218 | 171 | 1.0 | | 1.0 | | 115.9 | 119.3 | 117.2 |
| < 10 cpd | 26 | 34 | 27 | 0.94 | (0.53–1.66) | 0.92 | (0.51–1.65) | 120.1 | 114.2 | 116.6 |
| 10–19 cpd | 79 | 60 | 70 | 1.63[a] | (1.10–2.41) | 1.06 | (0.72–1.56) | 114.8 | 115.1 | 113.6 |
| 20–29 cpd | 143 | 122 | 121 | 1.46[a] | (1.05–2.03) | 1.11 | (0.80–1.53) | 118.1 | 116.9 | 117.0 |
| 30–39 cpd | 23 | 32 | 48 | 0.95 | (0.52–1.73) | 0.45[(a)] | (0.26–0.77) | 117.0 | 118.7 | 119.4 |
| ≥ 40 cpd | 28 | 21 | 40 | 1.77 | (0.94–3.34) | 0.66 | (0.39–1.11) | 117.0 | 109.2 | 113.6 |
| *P*-value for trend[c] | | | | *P* = 0.02 | | [*P* = 0.16] | | | | |
| Ex-smoker | 43 | 51 | 47 | 0.99 | (0.62–1.58) | 0.90 | (0.57–1.42) | 121.7 | 119.0 | 119.4 |
| Smoking status n/k | 29 | 17 | 30 | | | | | 118.0 | 116.2 | 113.7 |
| Total | 555 | 555 | 554 | | | | | | | |
| **Mothers** | | | | | | | | | | |
| ≥ 40 cpd | 28 | 21 | 40 | 1.77 | (0.94–3.34) | 0.66 | (0.39–1.11) | 117.0 | 109.2 | 113.6 |
| Lifelong non-smoker | 283 | 316 | 234 | 1.0 | | 1.0 | | 118.9 | 118.7 | 118.8 |
| < 10 cpd | 46 | 30 | 43 | 1.77[a] | (1.07–2.92) | 0.87 | (0.54–1.39) | 119.2 | 114.4 | 121.5 |
| 10–19 cpd | 114 | 88 | 100 | 1.51[a] | (1.08–2.13) | 0.95 | (0.69–1.31) | 115.1 | 114.0 | 113.4 |
| 20–29 cpd | 78 | 74 | 103 | 1.22 | (0.86–1.74) | 0.64[(a)] | (0.45–0.91) | 114.2 | 113.9 | 113.6 |
| ≥ 30 cpd | 7 | 14 | 36 | 0.48 | (0.17–1.37) | 0.18[(b)] | (0.08–0.40) | 98.0 | 121.2 | 111.6 |
| *P*-value for trend[c] | | | | *P* = 0.53 | | [*P* < 0.001] | | | | |
| Ex-smoker | 21 | 27 | 31 | 0.89 | (0.49–1.62) | 0.58 | (0.32–1.05) | 117.7 | 127.0 | 120.4 |
| Total | 549 | 549 | 547 | | | | | | | |

[a]*P* < 0.05; [b]*P* < 0.001, () indicates deficit; [c]two-tailed *P*-value, [] indicates negative trend; cpd = cigarettes per day

**Table 2** Childhood cancer risks by maternal cigarette smoking habits during the fifth month of pregnancy: IRESCC data, 1980–1983 diagnoses

| Maternal smoking habit | Cases | GP controls | Hospital controls | Childhood cancer risk: Cases vs GP controls | | Cases vs Hospital controls | | Mean birthweight (ounces) | | |
|---|---|---|---|---|---|---|---|---|---|---|
| | (*n*) | (*n*) | (*n*) | RR | (95% CI) | RR | (95% CI) | Cases | GP controls | Hospital controls |
| Not smoking | 354 | 383 | 331 | 1.0 | | 1.0 | | 119.0 | 118.9 | 118.6 |
| < 10 cpd | 46 | 34 | 30 | 1.49 | (0.93–2.39) | 1.44 | (0.88–2.34) | 118.2 | 116.7 | 118.7 |
| 10–19 cpd | 92 | 66 | 84 | 1.58[a] | (1.09–2.30) | 1.05 | (0.76–1.45) | 110.5 | 112.8 | 113.6 |
| 20–29 cpd | 49 | 54 | 72 | 1.02 | (0.68–1.54) | 0.65[(a)] | (0.44–0.96) | 116.3 | 111.9 | 110.9 |
| ≥ 30 cpd | 8 | 12 | 30 | 0.74 | (0.30–1.83) | 0.26[(b)] | (0.12–0.57) | 108.1 | 118.1 | 111.8 |
| P-value for trend[c] | | | | *P* = 0.36 | | [*P* = 0.003] | | | | |
| Total | 549 | 549 | 547 | | | | | | | |

[a]*P* < 0.05; [b]*P* < 0.001, () indicates deficit; [c]two-tailed *P*-value, [] indicates negative trend maternal smoking habit when cases were compared with hospital controls (not shown in Table).

The role of the source (mother or father) of paternal smoking data was investigated by analysing the paternal smoking data simultaneously with a binary variable indicating the presence or absence of the father at the interview and with interaction terms for smoking levels and presence of the father. A significant positive trend ($P = 0.03$) remained for the main effects of paternal smoking and childhood cancer risk when cases were compared with GP controls.

Information on the reliability of the smoking data was sought from a separate examination of the data relative to birth weights. Maternal smoking is known, from other sources, to produce low birth weights (US Department of Health and Human Services, 1980). Mean birth weights, by level of parental cigarette consumption, are also shown in Table 1. There were negative trends between birth weight and maternal daily consumption of cigarettes for cases ($P = 0.005$), GP controls ($P = 0.013$) and hospital controls ($P = 0.008$); similar trends were not found for paternal smoking habits. The effects of case/control status (three levels), maternal use of cigarettes (six levels), and paternal use of cigarettes (eight levels) on birth weight were examined in an analysis of variance. Only maternal consumption of cigarettes made a statistically significant contribution ($P < 0.001$) to explaining the variance in the birth weight variable. Maternal smoking habits at the fifth month of the pregnancy explained more of the variance in the birth weight variable than did maternal smoking habits before the pregnancy.

Relative risks for all types of childhood cancers combined are shown by maternal cigarette smoking habits during the pregnancy (fifth month of the pregnancy) in Table 2. The style of presentation follows that shown in Table 1. A non-significant positive trend

© 2001 Cancer Research Campaign 

**Table 3** Site-specific childhood cancer risks by parental cigarette smoking habits before the pregnancy (time of conception): IRESCC data, 1980–1983 diagnoses

| Parental smoking habit | ALL Cases (*n*) | ALL GP controls (*n*) | ALL RR | ALL 95% CI | Other RES neoplasms Cases (*n*) | Other RES neoplasms GP controls (*n*) | Other RES neoplasms RR | Other RES neoplasms 95% CI | CNS tumours Cases (*n*) | CNS tumours GP controls (*n*) | CNS tumours RR | CNS tumours 95% CI | Other childhood cancers Cases (*n*) | Other childhood cancers GP controls (*n*) | Other childhood cancers RR | Other childhood cancers 95% CI |
|---|---|---|---|---|---|---|---|---|---|---|---|---|---|---|---|---|
| **Fathers** | | | | | | | | | | | | | | | | |
| Lifelong non-smoker | 57 | 64 | 1.0 | | 29 | 41 | 1.0 | | 29 | 23 | 1.0 | | 69 | 90 | 1.0 | |
| < 10 cpd | 7 | 9 | 0.99 | 0.35–2.85 | 6 | 7 | 1.32 | 0.32–5.51 | 2 | 4 | 0.51 | 0.09–2.97 | 11 | 14 | 1.13 | 0.46–2.80 |
| 10–19 cpd | 18 | 16 | 1.34 | 0.62–2.91 | 11 | 8 | 2.65 | 0.83–8.46 | 12 | 9 | 1.25 | 0.42–3.72 | 38 | 27 | 1.81 | 0.79–2.30 |
| 20–29 cpd | 36 | 35 | 1.32 | 0.72–2.45 | 33 | 17 | 3.69[b] | 1.49–9.15 | 17 | 20 | 0.52 | 0.21–1.33 | 57 | 50 | 1.55 | 0.91–2.65 |
| 30–39 cpd | 9 | 5 | 2.33 | 0.71–7.63 | 2 | 7 | 0.29 | 0.03–2.56 | 1 | 5 | 0.15 | 0.01–1.54 | 11 | 15 | 1.01 | 0.42–2.46 |
| ≥ 40 cpd | 12 | 3 | 5.29 | 1.31–21.30 | 4 | 6 | 1.20 | 0.29–5.05 | 5 | 4 | 0.64 | 0.08–4.79 | 7 | 8 | 1.39 | 0.46–4.21 |
| *P*-value for trend[c] | | | *P* = 0.06 | | | | *P* = 0.35 | | | | [*P* = 0.67] | | | | *P* = 0.11 | |
| Ex-smoker | 8 | 15 | 0.59 | 0.23–1.49 | 5 | 7 | 0.98 | 0.25–3.92 | 5 | 11 | 0.28 | (0.07–1.17) | 25 | 18 | 1.80 | 0.89–3.63 |
| Smoking status n/k | 2 | 2 | | | 6 | 3 | | | 7 | 2 | | | 14 | 10 | | |
| **Mothers** | | | | | | | | | | | | | | | | |
| Lifelong non-smoker | 85 | 86 | 1.0 | | 48 | 54 | 1.0 | | 32 | 40 | 1.0 | | 118 | 136 | 1.0 | |
| < 10 cpd | 9 | 7 | 1.34 | 0.46–3.87 | 9 | 8 | 1.20 | 0.41–3.47 | 11 | 2 | 6.56[a] | 1.36–31.73 | 17 | 13 | 1.61 | 0.72–3.59 |
| 10–19 cpd | 24 | 23 | 1.11 | 0.59–2.08 | 22 | 12 | 2.81[a] | 1.07–7.39 | 16 | 16 | 1.28 | 0.55–3.03 | 52 | 37 | 1.81[a] | 1.04–3.14 |
| 20–29 cpd | 21 | 22 | 0.98 | 0.51–1.85 | 15 | 13 ] | 1.38 | 0.58–3.26 | 13 | 14 ] | 1.30 | 0.52–3.22 | 29 | 25 | 1.42 | 0.79–2.58 |
| ≥ 30 cpd | 1 | 4 | 0.26 | 0.03–2.38 | 1 | 4 ] | | | 0 | 0 ] | 0 | | 5 | 6 | 1.01 | 0.26–3.93 |
| *P*-value for trend[c] | | | [*P* = 0.56] | | | | *P* = 0.36 | | | | *P* = 0.71 | | | | *P* = 0.39 | |
| Ex-smoker | 8 | 6 | 1.44 | 0.48–4.31 | 1 | 5 | 0.21 | 0.02–1.77 | 4 | 4 | 1.50 | 0.27–8.51 | 8 | 12 | 0.81 | 0.32–2.02 |

[a]$P < 0.05$; [b]$P < 0.01$, () indicates deficit; [c]two-tailed *P*-value, [] indicates negative trend

*© 2001 Cancer Research Campaign*

($P = 0.36$) is shown for smoking habit and childhood cancer risk when cases are compared with GP controls, albeit there is a significantly elevated point estimate of relative risk for an intermediate smoking category (10–19 cpd, RR = 1.58). A highly significant negative trend ($P = 0.003$) is shown for the corresponding analysis with hospital controls, with significantly depressed relative risks for the two highest smoking categories (20–29 cpd, RR = 0.65; ≥ 30 cpd, RR = 0.26). Additional adjustment for maternal age at the birth of the survey child, socioeconomic category and ethnic origin had little material effects on these two sets of relative risks.

The percentages of survey parents who were current smokers before the pregnancy were compared with national (expected) percentages obtained from the General Household Surveys (OPCS, 1980; 1990), adjusting for sex, age at time of conception (16–19, 20–24, 25–34, 35–59, ≥ 60) and year of conception (2-year intervals). Observed and expected percentages of smokers in case fathers were 56.8% and 51.1% respectively ($P < 0.01$). Corresponding percentages for other parents were as follows: GP control fathers 50.0% and 51.5%; hospital control fathers 58.4% and 50.7% ($P < 0.001$); case mothers 44.6% and 44.4%; GP control mothers 37.5% and 44.6% ($P < 0.001$); hospital control mothers 51.0% and 44.3% ($P < 0.01$).

Relative risks for four types of childhood cancer (acute lymphoblastic leukaemia (ALL), other neoplasms of the reticulo-endothelial system, tumours of the central nervous system, all other cancers) are shown by parental cigarette habits before the pregnancy in Table 3. These relative risks relate to case/GP control comparisons and are based on separate analyses of paternal and maternal habits. The trend in the risk of ALL with paternal smoking habit approached statistical significance ($P = 0.06$).

### DISCUSSION

Confident interpretation of these data is difficult in that the two sets of controls produced very different findings: the analyses with GP controls supported the hypothesis under test, the analyses with hospital controls did not. It was intended from the outset to give more weight to the analyses with GP controls because these were population-based, though it was not intended to ignore the analyses with hospital controls. The comparisons with national data from the General Household Surveys suggest, however, that there was an unusually high prevalence of smokers in the hospital control parents, and it may well be that as far as smoking is concerned, the hospital control parents in this study are not a representative sample of the population at risk. The highly significant negative trends shown for childhood cancer risks and maternal smoking habits when cases were compared with hospital controls, trends which receive no support from the fairly extensive epidemiological literature on maternal smoking and childhood cancer risks, would support such an evaluation. It is possible that the unusually high prevalence of smokers in the hospital control parents reflects a tendency for parents who accept risks for themselves by smoking to allow their children to take risks (activities leading to hospital admission for accidents).

The analyses with GP controls provide further supportive evidence of an association between the daily cigarette smoking habits of fathers and cancer in their offspring; there was no significant trend with maternal habit though significant relative risks were shown for lighter smokers. However, the smoking of cigarettes by mothers can, with some confidence, be excluded as an important risk factor for the generality of childhood cancers because the prevalence of smoking in case mothers (44.6%) was very similar to that in the general population (44.4%) (see also Sorahan et al, 1997*a*: Thornton and Lee, 1998; Sasco and Vainio, 1999).

If the paternal smoking association is causal in nature, this might be due either to pre-conception effects or to the effects of passive smoking on young infants, or both. A passive smoking effect seems unlikely because of the weight of evidence against maternal smoking being a risk factor for childhood cancers; it might be imagined that, in general, the infant has more contact with passive smoke from the mother than from the father. A pre-conception effect is not biologically implausible and evidence for potential mechanisms has been reviewed (Wyrobek, 1993; Wyrobek and Adler, 1996; Woodall and Ames, 1997).

The paternal results are unlikely to be a chance finding because in each of the three other relevant UK studies (OSCC), trends with smoking habit have been highly significant ($P < 0.001$). Confounding also presents an unlikely sole explanation. The potential confounders which have been considered with these data (socioeconomic category, ethnic origin, age of father) had little effect on the paternal smoking findings and the use of alcohol can be excluded on the basis of previous work (Sorahan et al, 1995; Ji et al, 1997). If an unknown variable was confounding the paternal smoking effect, it would need, by definition, to be associated with higher risks than paternal smoking, both for point estimates of relative risk and for attributable risk; an unusual occupational exposure would not, therefore, provide a likely candidate.

One key issue in evaluating the importance of these findings is the reliability of IRESCC data. For the data relating to mothers' smoking habits there was one test of their reliability, namely the relation with birth weight. For the fathers' smoking habits there was no comparable test, though the comparison of national smoking prevalence data with data for GP control fathers was reassuring and did not suggest that the paternal smoking effect was an artifact caused by the GP control fathers having an unusually low prevalence of smokers. The possibility of differential reporting of cigarette smoking habits between case and control fathers remains, although it has been possible in this study to eliminate the source (mother or father) of the paternal data as an explanation for the positive findings.

This study has a number of advantages over the earlier OSCC reports. It comprises incident cancers rather than cancer deaths and considerable efforts were made to interview case parents soon after the case diagnoses. Consequently the paternal findings in this study cannot merely reflect inaccurate recall in the timing of any changes in smoking brought on by the death of a child or be explained by paternal smoking only increasing mortality rates in children diagnosed with cancer. Participation rates were also better than for the later OSCC reports, although as with all case-control studies the effects of having to ignore the non-responders are not known. Caution is still required in interpreting these findings because they are based on fairly small numbers of cases and controls (given the size of the relative risks being evaluated) and it is not possible to exclude all potential biases as the cause of the positive findings.

More information is required. There are two recent case-control reports from the US Children's Cancer Group (Brondum et al, 1999; Wen et al, 2000). These studies have considerable overlap in membership of the case series. The first report considered 2359 cases of acute childhood leukaemia diagnosed in the period 1989–1993 and found no association with paternal smoking after

© 2001 Cancer Research Campaign adjusting for paternal race, paternal education and family income (RR = 1.01, 95% CI = 0.91–1.13) (present authors' calculation) (Brondum et al, 1999). The second report considered 2343 cases of acute childhood leukaemia diagnosed in the period 1983–1993 and found a significant association with paternal smoking unadjusted for other variables (RR = 1.2, $P = 0.04$) (Wen et al, 2000). A recent report from a similarly large German case-control study considered 2358 cases of childhood cancer but no associations were seen for paternal smoking habits in the 3 months before pregnancy (Schüz et al, 1999). The new, large UK case-control study investigating the aetiology of childhood cancer is also expected to evaluate the role of paternal smoking. Analyses of cancer in the offspring of subjects whose smoking habits were collected in contexts other than case-control studies would also be helpful.

### ACKNOWLEDGEMENTS

We again thank the parents of the children included in the study and the many general practitioners, consultants and nurses in the three regions who made the study possible. Earlier financial support was provided by the Cancer Research Campaign, the Leukaemia Research Fund, the Department of Health and Social Security, the Scottish Home and Health Department, the Special Trustees for the former United Birmingham Hospitals Trust Funds, and the Special Trustees of Leeds Western Health Authority. Earlier assistance with photocopying, microfilming and computing was supplied by Rank Xerox, Bell and Howell and Systime Ltd. Financial support for these analyses was provided by Departmental reserves maintained by TS. We thank Jaswant Bal for the re-abstraction of data. We thank Drs Gerald Draper, Ann Hartley, Paul Hopton and John Waterhouse for their earlier contributions to the IRESCC.

### REFERENCES

Birch JM, Mann JR, Cartwright RA, Draper GJ, Waterhouse JAH, Hartley AL, Johnston HE, McKinney PA, Stiller CA and Hopton PA (1985) The Inter-regional epidemiological study of childhood cancer (IRESCC). Study design, control selection and data collection. *Br J Cancer* **52**: 915–922

Birch JM, Hartley AL, Teare MD, Blair V, McKinney PA, Mann JR, Stiller CA, Draper GJ, Johnston HE, Cartwright RA and Waterhouse JAH (1990) The Inter-regional epidemiological study of childhood cancer (IRESCC): case control study of children with central nervous system tumours. *Br J Neurosurgery* **4**: 17–26

Brondum J, Shu XO, SteinBUCH M, Severson RK, Potter JD and Robison LL (1999) Parental cigarette smoking and the risk of acute leukaemia in children. *Cancer* **85**: 1380–1388

Cartwright RA, McKinney PA, Hopton PA, Birch JM, Hartley AL, Mann JR, Waterhouse JAH, Johnston HE, Draper GJ and Stiller C (1984) Ultrasound examinations in pregnancy and childhood cancer. *Lancet* **ii**: 999–1000

Hartley AL, Birch JM, McKinney PA, Blair V, Teare MD, Carrette J, Mann JR, Stiller CA, Draper GJ, Johnston HE, Cartwright RA and Waterhouse JAH (1988*a*) The Inter-regional epidemiological study of childhood cancer (IRESCC): past medical history in children with cancer. *J Epidemiol Commun Health* **42**: 235–242

Hartley AL, Birch JM, McKinney PA, Teare MD, Blair V, Carrette J, Mann JR, Draper GJ, Stiller CA, Johnston HE, Cartwright RA and Waterhouse JAH (1988*b*) The inter-regional epidemiological study of childhood cancer (IRESCC): case control study of children with bone and soft tissue sarcomas. *Br J Cancer* **58**: 838–843

Hopton PA, McKinney PA, Cartwright RA, Mann JR, Birch JM, Hartley AL, Waterhouse JAH, Johnston HE, Draper GJ and Stiller CA (1985) X-rays in pregnancy and the risk of childhood cancer. *Lancet* **ii**: 773

Ji BT, Shu XO, Linet MS, Zheng W, Wacholder S, Gao YT, Ying DM and Jin F (1997) Paternal pre-conception cigarette smoking and the risk of childhood cancer in the offspring of nonsmoking mothers. *J Natl Cancer Inst* **89**: 238–244

Johnston HE, Mann JR, Williams J, Waterhouse JAH, Birch JM, Cartwright RA, Draper GJ, Hartley AL, McKinney PA, Hopton PA and Stiller CA (1986) The Inter-regional epidemiological study of childhood cancer (IRESCC): case-control study in children with germ cell tumours. *Carcinogenesis* **7**: 717–722

Mann JR, Dodd HE, Draper GJ, Waterhouse JAH, Birch JM, Cartwright RA, Hartley AL, McKinney PA and Stiller CA (1993) Congenital abnormalities in children with cancer and their relatives: results from a case-control study (IRESCC): *Br J Cancer* **68**: 357–363

McKinney PA and Stiller CA (1986) Maternal smoking during pregnancy and the risk of childhood cancer. *Lancet* **ii**: 519

McKinney PA, Cartwright RA, Stiller CA, Hopton PA, Mann JR, Birch JM, Hartley AL, Draper GJ, Waterhouse JAH and Johnston HE (1985) Inter-regional epidemiological study of childhood cancer (IRESCC). Childhood cancer and the consumption of debendox and related drugs in pregnancy. *Br J Cancer* **52**: 923–929

McKinney PA, Cartwright RA, Saiu JMT, Mann JR, Stiller CA, Draper GJ, Hartley AL, Hopton PA, Birch JM, Waterhouse JAH and Johnston HE (1987) The Inter-regional epidemiological study of childhood cancer (IRESCC): a case control study of aetiological factors in leukaemia and lymphoma. *Arch Dis Childhood* **62**: 279–287

Office of Population Censuses and Surveys (1980) *General Household Survey 1978*. Series GHS no 8. HMSO: London

Office of Population Censuses and Surveys (1990) *General Household Survey 1988*. Series GHS no 19. HMSO: London

Sasco AJ and Vainio H (1999) From *in utero* and childhood exposure to parental smoking to childhood cancer: a possible link and the need for action. *Human Exp Toxicol* **15**: 192–201

Schüz J, Kaatsch P, Kaletsch U, Meinert R and Michaelis J (1999) Association of childhood cancer with factors related to pregnancy and birth. *Int J Epidemiol* **28**: 631–639

Sorahan T, Lancashire R, Prior P, Peck I and Stewart A (1995) Childhood cancer and parental use of alcohol and tobacco. *Ann Epidemiol* **5**: 354–359

Sorahan T, Lancashire R, Hulten MA, Peck I and Stewart AM (1997*a*) Childhood cancer and parental use of tobacco: deaths from 1953 to 1955. *Br J Cancer* **75**: 134–138

Sorahan T, Prior P, Lancashire RJ, Faux SP, Hulten MA, Peck IM and Stewart AM (1997*b*) Childhood cancer and parental use of tobacco: deaths from 1971 to 1976. *Br J Cancer* **76**: 1525–1531

Thornton AJ and Lee PN (1998) Parental smoking and risk of childhood cancer: A review of the evidence. *Indoor Built Environ* **7**: 65–86

US Department of Health and Human Services (1980) *The health consequences of smoking for women: a report of the Surgeon General*. US Department of Health and Human Services, Public Health Service, Office of the Assistant Secretary for Health, Office on Smoking and Health: Washington, DC

Wen WQ, Shu XO, Steinbuch M, Severson RK, Reaman GH, Buckley JD and Robison LL (2000) Paternal military service and risk for childhood leukaemia in offspring. *Am J Epidemiol* **151**: 231–240

Woodall AA and Ames BN (1997) Nutritional prevention of DNA damage to sperm and consequent risk reduction in birth defects and cancer in offspring. In *Preventive nutrition: The comprehensive guide for health professionals*. Bendich A and Deckelbaum RJ (eds) Humana Press Inc: Totowa, New Jersey

Wyrobek AJ (1993) Methods and concepts in detecting abnormal reproductive outcomes of paternal origin. *Reprod Toxicol* **7** (suppl 1): 3–16

Wyrobek AJ and Adler ID (1996) Detection of aneuploidy in human and rodent sperm using FISH and applications of sperm assays of genetic-damage in heritable risk-evaluation. *Mutat Res* **352**: 173–179

© 2001 Cancer Research Campaign
